# Biofilm Control with Rare-Earth Oxides: A Mechanistic Framework for Next-Generation Antibiofilm Materials

**DOI:** 10.3390/nano16050302

**Published:** 2026-02-27

**Authors:** John H. T. Luong

**Affiliations:** School of Chemistry, University College Cork, T12 YN60 Cork, Ireland; luongprof@gmail.com

**Keywords:** antibiofilm, rare earth oxides, nanoparticles, cerium oxides, lanthanum oxide, samarium oxide, reactive oxygen species, antimicrobial, cytotoxicity

## Abstract

Biofilm-associated infections remain a major barrier to wound healing, implant integration, and chronic infection management. Rare-earth oxides (REOs) have emerged as promising antibiofilm materials, though their mechanisms, limitations, and translational potential are still being defined. Cerium oxide (CeO_2_) serves as the benchmark due to its redox adaptability, oxygen-vacancy-driven catalytic activity, and host compatibility. In contrast, non-ceria REOs show antibiofilm effects under more restricted conditions, often requiring surface functionalization, composite architectures, or hybrid organic–inorganic interfaces—such as polyphenol coatings or hydroxyapatite-based composites—to achieve comparable activity. Across systems, biofilm control arises not from bactericidal potency but from matrix-level mechanisms including extracellular polymeric substance (EPS) destabilization, extracellular DNA (eDNA) sequestration, redox modulation, and quorum-sensing interference. Preclinical and near-clinical evidence, particularly in chronic wound models, supports the translational relevance of these mechanisms, though the evidence base remains preliminary. This review synthesizes mechanistic data across cerium-, samarium-, lanthanum-, and strontium-based systems to establish a unified framework for REO-mediated biofilm disruption. REOs are positioned as biofilm-modulating platforms that complement antibiotics, enhance healing, and improve outcomes. Design rules emphasize controlled redox activity, targeted coordination chemistry, functional surface engineering, and host-compatible performance, alongside regulatory and manufacturing guidance for future development.

## 1. Introduction

Biofilms represent the predominant mode of bacterial growth in natural, industrial, and clinical settings, and their intrinsic tolerance to antimicrobial agents remains one of the most persistent challenges in infection management. In contrast to planktonic cells, biofilm-associated bacteria are embedded within a self-produced extracellular polymeric substance (EPS) matrix composed of polysaccharides, proteins, lipids, and extracellular DNA (eDNA). This matrix functions as a physical diffusion barrier and a biochemical shield, sequestering antimicrobials, modulating local pH, and buffering oxidative or chemical stressors [1].

Crucially, antibiotic failure against biofilms cannot be attributed solely to impaired penetration. Mature biofilms exhibit pronounced physiological and metabolic heterogeneity, including nutrient-limited dormancy, altered redox states, and quorum-regulated phenotypes that collectively reduce susceptibility to antibiotic killing [2,3]. These adaptations allow biofilm-embedded cells to survive concentrations of antibiotics that would be lethal to their planktonic counterparts.

Reactive oxygen species (ROS) play a central role in the bactericidal activity of many conventional antibiotics. However, biofilms possess robust antioxidant defenses that neutralize ROS before they inflict lethal damage. These include intracellular redox buffers (e.g., glutathione, catalase, and peroxidases) and EPS-associated scavenging mechanisms that collectively suppress oxidative stress [4]. As a result, even ROS-generating nanomaterials—highly effective against pathogens with simpler or less protective biofilms—show markedly reduced activity against organisms such as *Pseudomonas aeruginosa*, whose EPS matrix and intrinsic redox defenses are exceptionally well developed [5].

The EPS of *P. aeruginosa* is a particularly formidable barrier, composed primarily of alginate, Pel, and Psl polysaccharides.

- Psl, a neutral, branched pentasaccharide of D-mannose, D-glucose, and L-rhamnose, plays a dominant role in early biofilm establishment by anchoring cells to surfaces and forming a scaffold-like architecture. Psl also contributes to immune evasion by inhibiting phagocytosis and suppressing neutrophil ROS production [6].
- Pel, a cationic polymer of 1–4 linked galactosamine and N-acetylgalactosamine, reinforces the structural integrity of mature biofilms and forms the characteristic pellicle at the air–liquid interface. Its positive charge enables strong binding to negatively charged eDNA and several classes of antibiotics, including aminoglycosides such as tobramycin.
- Alginate, an anionic exopolysaccharide overproduced in mucoid isolates, contributes to chronic infection persistence by enhancing mechanical stability and scavenging ROS.

**Figure 1:**
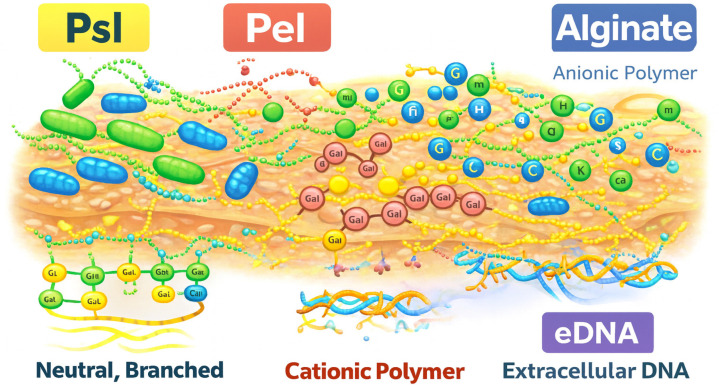
The biofilm of *P. aeruginosa* consists of Pel, a positively charged extracellular polysaccharide. Its cationic nature enables strong electrostatic interactions with negatively charged extracellular DNA (eDNA) and antimicrobial agents, reinforcing biofilm structural integrity and contributing to antimicrobial tolerance. Each panel highlights the molecular components most relevant to REO interactions, including eDNA, polysaccharides, and redox-active microenvironments, thereby providing the mechanistic foundation for later sections. (Created with BioRender.com; original artwork by the author).

Combined Pel, Psl, and alginate create a chemically and physically resilient matrix that restricts antimicrobial penetration, neutralizes ROS, and shields embedded bacteria from host immune factors [5,6,7] (Figure 1).

Within this evolving landscape, nanomaterials have emerged as particularly compelling tools. Their physicochemical properties enable interactions with biofilms that are fundamentally distinct from those of molecular antibiotics, including catalytic ROS generation [8,9,10,11,12], EPS depolymerization [5,6], membrane disruption, and interference with microbial communication. However, such mechanisms vary widely across material classes and must be attributed cautiously to avoid overgeneralization. REO nanomaterials, particularly CeO_2_, have drawn increasing interest due to their tunable redox activity, oxygen-vacancy chemistry, and affinity for biomolecular functional groups, while evidence for intrinsic antibiofilm activity in non-ceria REOs remains comparatively sparse. Where activity has been reported for non-ceria systems, it often occurs under specific functionalized or composite configurations; however, this reflects limited available studies rather than evidence that unmodified particles lack intrinsic activity.

While numerous studies have documented individual REO antimicrobial or catalytic behavior, no prior review has integrated these findings into a unified, mechanism-oriented framework. The present review aims to synthesize dispersed evidence into a coherent structure, distinguishing established mechanisms from hypothesized or emerging concepts. Accordingly, this review critically examines established and emerging mechanisms of REO-mediated biofilm disruption, differentiates evidence-supported interactions from hypothesized parallels, and integrates findings across cerium-, samarium-, lanthanum-, and selected strontium-based systems into a unified mechanistic framework.

## 2. Physicochemical Foundations for the Antibiofilm Activity of REOs

REOs are nanomaterials with unique catalytic, electronic, and coordination properties due to their partially filled 4f orbitals. Nanoscale REOs exhibit high surface-to-volume ratios, tunable oxidation states, and a strong propensity for defect formation—particularly oxygen vacancies. These features distinguish REOs from classical antimicrobial metal oxides such as silver, copper, or zinc oxide, whose biological activity is dominated by ion release and nonspecific cytotoxicity [8]. In contrast, REOs interact with biofilms through catalytic, coordination-driven, and surface-interactive mechanisms that differ substantially from conventional antimicrobial pathways and cannot be reduced solely to oxidative toxicity.

### 2.1. Redox Activity and Oxygen-Vacancy Chemistry

Among REOs, cerium oxide (CeO_2_) is the prototypical redox-active material. CeO_2_ nanoparticles can reversibly switch between Ce^3+^ and Ce^4+^ oxidation states, a process facilitated by oxygen vacancies that act as electron reservoirs. This redox plasticity enables CeO_2_ to function as a nanozyme, catalyzing superoxide dismutation, hydrogen peroxide decomposition, and peroxidase-like reactions depending on local pH and redox conditions [9,10]. In biofilm microenvironments, characterized by steep redox gradients, hypoxia, and abundant EPS-associated antioxidants, the catalytic behavior of CeO_2_ becomes particularly relevant. Oxygen vacancies enhance electron transfer and ROS modulation, allowing CeO_2_ to either amplify oxidative stress or buffer it, depending on the biochemical milieu [11]. Defect formation, particularly oxygen vacancies, which underpin CeO_2_’s redox-switching behavior, is central to REO catalytic activity [11,12]. Because these vacancies directly influence the balance between Ce^3+^ and Ce^4+^, their density can be deliberately tuned through hydrothermal synthesis conditions, aliovalent dopant incorporation (e.g., Gd^3+^ and Sm^3+^), or controlled annealing atmospheres, each shown to shift catalytic activity profiles in biological systems [11,12]. This dynamic redox behavior is distinct from the static ROS-generation profiles of TiO_2_ or ZnO nanoparticles, which require external stimuli such as UV light.

Other mixed-valence REOs, including praseodymium oxide (Pr_6_O_11_) and terbium oxide (Tb_4_O_7_), also exhibit oxygen-vacancy-driven catalytic activity, though their biological applications remain less explored [12]. Variations in valence-state cycling, oxygen-vacancy density, and surface coordination chemistry strongly influence how metal-oxide systems modulate redox processes in biological environments. Studies on advanced catalytic and immunomodulatory nanomaterials [13,14] show that materials with mixed-valence states, such as Pr- and Tb-based oxides, exhibit redox behaviors distinct from CeO_2_, including altered vacancy formation energies, different electron-transfer efficiencies, and shifts in catalytic selectivity. These principles support the expectation that non-ceria REOs will display mechanistically divergent redox-modulation profiles compared with CeO_2_ in biological systems. These findings indicate that while multiple REOs exhibit mixed-valence chemistry, CeO_2_ remains uniquely adaptable in its ability to modulate ROS across diverse biochemical conditions.

### 2.2. Redox-Inactive REOs: Coordination Chemistry and Surface Interactions

**Figure 2:**
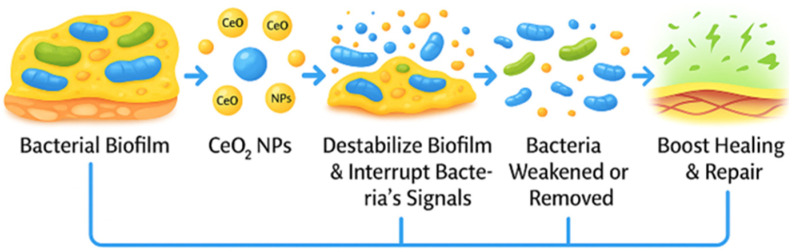
CeO_2_-mediated biofilm disruption and tissue repair. CeO_2_ nanoparticles interact with bacterial biofilms, destabilizing the EPS matrix and interfering with quorum-sensing signals. This disruption weakens or dislodges embedded bacteria, enabling their removal and facilitating subsequent tissue healing and repair. (Created with BioRender.com; original artwork by the author).

As illustrated in Figure 2, redox-inactive rare-earth oxides primarily interact with biofilms through coordination chemistry and surface-mediated mechanisms, including binding to extracellular polymeric substances, modulation of adhesion, and disruption of biofilm structural integrity.

Besides CeO_2_, lanthanum oxide (La_2_O_3_), samarium oxide (Sm_2_O_3_), and strontium-containing oxides, which are chemically distinct from lanthanide REOs. They are largely redox-inactive under physiological conditions, and their antibiofilm activity arises primarily from surface-mediated physicochemical interactions rather than redox cycling. In biofilm environments, these rare-earth oxides interact directly with EPS and microbial membranes through several complementary mechanisms:

Electrostatic affinity for eDNA. Trivalent REO cations such as La^3+^ and Sm^3+^ exhibit a strong affinity for phosphate groups in eDNA, enabling condensation and partial neutralization of the eDNA network, which weakens EPS cohesion and cation-dependent cross-linking [15].

Competition with biologically essential cations. Due to their high charge density and Lewis acidity, lanthanide ions can competitively interact with phosphate- and carboxylate-containing biomolecules. Computational studies suggest that La^3+^ may interact strongly with biomolecular coordination sites; however, these findings should not be interpreted as direct evidence that lanthanides universally outcompete Ca^2+^ or Mg^2+^ in biological environments [16].

Membrane perturbation. Trivalent lanthanides can interact with phospholipid head groups and alter local membrane organization; however, direct experimental evidence for REO nanoparticles inducing such effects in live bacterial membranes remains limited.

Lewis-acid interactions and EPS destabilization. The strong Lewis acidity of REO surfaces can weaken polysaccharide cross-linking or promote hydrolytic destabilization of EPS components, particularly in matrices rich in phosphate and carboxylate functionalities.

Overall, available evidence indicates the potential for these interactions, but systematic REO-specific studies are limited, and mechanistic conclusions must be framed accordingly.

### 2.3. Nanostructure, Surface Charge, and Curvature-Dependent Interactions

Beyond redox and coordination chemistry, REO antibiofilm activity is influenced by several nanoscale structural features: (i) particle size and curvature, (ii) surface charge (zeta potential), and (iii) hydration layers and surface hydroxylation.

First, nanoparticles with diameters below ~10 nm can penetrate EPS channels and act as nano-wedges, mechanically disrupting matrix cohesion. High curvature increases surface reactivity and exposes a greater density of coordination sites [17,18]. Second, surface charge governs interactions with negatively charged EPS components such as eDNA, alginate, and Psl. Positively charged REOs exhibit enhanced penetration and binding within biofilms. Finally, REOs form structured hydration shells that influence colloidal stability and biomolecular interactions. Surface hydroxyl groups enable hydrogen bonding with EPS polysaccharides, further modulating adhesion and penetration.

### 2.4. Distinctiveness of REOs Among Antimicrobial Nanomaterials

Compared with conventional metal oxides such as Ag_2_O, CuO, and ZnO, REOs represent a mechanistically distinct class of antibiofilm materials. While traditional oxides primarily rely on ion release and nonspecific oxidative toxicity, REOs operate through catalytic, coordination-driven, and surface-mediated mechanisms. Their distinguishing features include lower cytotoxicity due to tightly bound ion release, catalytic rather than consumptive redox behavior, and strong affinity for phosphate- and carboxylate-rich biomolecules. REOs also offer tunability through oxygen-vacancy and defect engineering, and they show potential for synergy with antibiotics by weakening biofilm architecture rather than directly killing bacteria. These attributes position REOs as promising, though still under-characterized, antibiofilm platforms whose activity must be evaluated within the limitations of currently available data.

### 2.5. Need for Comparative, Mechanism-Focused Studies

Despite their promise, the literature on REO antibiofilm activity remains uneven, with most studies focused on CeO_2_ and relatively few examining La_2_O_3_, Sm_2_O_3_, or mixed-valence REOs. The diversity of REO physicochemical properties underscores the urgent need for systematic, comparative studies that evaluate how redox behavior, surface chemistry, coordination strength, and defect structure correlate with antibiofilm outcomes. An additional physicochemical factor relevant to REO–biofilm interactions is the relationship between ionic size, charge density, and surface reactivity. The lanthanide series exhibits a well-defined contraction in ionic radii, which influences coordination geometry and ligand affinity.

However, ionic radii alone do not determine binding affinity in hydrated biological systems, and such extrapolations must be framed cautiously. Studies on oxidized nanomaterials demonstrate that surface oxidation can drastically alter membrane interactions and cytotoxicity [19]. Although these studies involve non-REO materials (e.g., graphene oxide), they highlight broad physicochemical principles but should not be interpreted as direct evidence of REO behavior without REO-specific validation.

## 3. Cerium Oxide as the Antibiofilm Benchmark Among REOs

CeO_2_ nanoparticles are the most extensively studied REOs in antibiofilm research and serve as the field’s benchmark. Their dominance stems from their unique redox adaptability, oxygen-vacancy–driven catalytic behavior, and strong biological compatibility—features that align with the physicochemical demands of mature biofilms. However, CeO_2_’s established status reflects the relative abundance of available data rather than definitive evidence that its mechanisms are universally superior to those of other REOs. Current literature supports redox-mediated EPS disruption and host-compatible interactions, but direct comparative evaluations across REOs remain limited.

### 3.1. Redox Cycling and Nanozyme Behavior

CeO_2_’s reversible Ce^3+^/Ce^4+^ cycling, enabled by oxygen vacancies, underlies its superoxide dismutase-, catalase-, and peroxidase-like nanozyme activity [9,10,11,12]. These vacancies act as electron reservoirs, allowing CeO_2_ to modulate ROS levels dynamically. Because biofilms contain steep and spatially heterogeneous redox gradients, nanoceria can modulate local oxidative conditions in a controlled manner, restoring redox balance without triggering the acute oxidative damage commonly associated with bactericidal metals [20,21]. This adaptive redox behavior distinguishes CeO_2_ from redox-inactive REOs; however, the extent to which such catalytic modulation translates into superior antibiofilm performance requires additional comparative validation, particularly under standardized conditions.

### 3.2. EPS and eDNA Destabilization

CeO_2_ also targets the biofilm matrix. Its strong affinity for eDNA, mediated by electrostatic and coordination interactions, loosens EPS structure and reduces cohesion [7,9,15]. Lanthanide coordination chemistry supports the plausibility of this mechanism; Ce^3+^ interacts strongly with phosphate groups, and computational studies indicate potential competitive behavior with biologically essential cations [16]. However, attributing direct “outcompetition” of Ca^2+^ or Mg^2+^ based solely on ionic radii oversimplifies biological binding equilibria. Ionic size data [18] provide useful context but should not be interpreted as quantitative predictors of binding affinity in hydrated, heterogeneous EPS environments. Surface oxidation state further modulates CeO_2_’s interactions with membranes and biomolecules [19]. These matrix-level effects occur across multiple species, supporting a broad-spectrum, structure-focused mechanism [22,23]. Nevertheless, systematic REO-specific studies comparing EPS affinity across materials remain scarce, and conclusions should be framed as supported trends rather than universal properties.

### 3.3. Disruption Versus Killing

Although CeO_2_ exhibits antibacterial activity, its antibiofilm effects primarily reflect structural disruption rather than direct killing. CeO_2_ reduces biomass, thickness, and cohesion even when bacterial viability is only moderately affected [24,25]. Nanoceria of 1–2 nm and 10–12 nm inhibits *S. aureus* and *P. aeruginosa* biofilms despite modest antimicrobial potency [24]. Unlike highly cytotoxic metal oxides (e.g., Ag_2_O and CuO), CeO_2_’s redox window allows modulation of oxidative stress without extensive collateral damage to host tissue [26]. This property supports its potential utility in chronic infection settings, although optimal dosing and long-term safety still require formal clinical evaluation.

### 3.4. Biofilm–Host Compatibility and Relevance to Chronic Wounds

CeO_2_’s dual ability to disrupt biofilms and support healing is frequently highlighted in chronic wound and diabetic foot ulcer research. A frequently cited 2019 case report describes topical nanoceria use in three diabetic foot ulcer patients [27]; although encouraging, this evidence should be interpreted as preliminary and hypothesis-generating rather than clinically definitive. More broadly, nanozyme-based cryogels show that redox-active materials can simultaneously target biofilms, oxidative stress, and inflammation in diabetic wounds [28]. Nanoceria’s antioxidant and anti-inflammatory effects further enhance angiogenesis and modulate cytokine signaling [29,30], highlighting its potential advantage over cytotoxic antimicrobial nanoparticles. However, the mechanistic parallels between cryogel systems and CeO_2_ require clearer qualification, given that cryogels may not contain REO components. Their relevance lies in demonstrating how catalytic redox materials—REO or otherwise—may influence infected wound environments.

### 3.5. Why Cerium Oxide Succeeds Where Other REOs Struggle

CeO_2_’s prominence reflects both its unique mixed-valence redox chemistry and the disproportionate volume of available studies. While Sm_2_O_3_, La_2_O_3_, and strontium-based oxides rely primarily on surface coordination interactions and often appear in functionalized or composite forms, the limited number of studies on unmodified non-ceria REOs prevents firm conclusions about their intrinsic antibiofilm potential. Current comparative analyses attribute CeO_2_’s performance to oxygen-vacancy–driven catalytic behavior [31,32,33], but direct, side-by-side evaluations across REOs are largely absent. Thus, CeO_2_ serves as the practical benchmark due to available evidence, rather than a proven superior agent across all contexts.

## 4. Non-Ceria REOs: Lessons from Partial Success

Although CeO_2_ dominates REO antibiofilm research, studies on samarium-, lanthanum-, and strontium-containing oxides reveal alternative, largely non-redox mechanisms of biofilm control. The literature on these materials remains sparse, and where activity has been observed, it frequently occurs in functionalized or composite systems. This pattern reflects limited investigation rather than conclusive evidence that intrinsic oxide chemistry is insufficient. Accordingly, performance differences should be interpreted as research gaps rather than mechanistic limitations.

**Figure 3:**
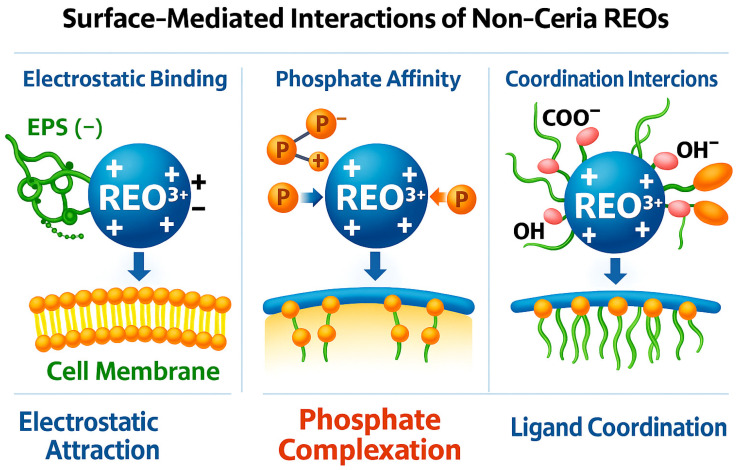
Three dominant interfacial processes are proposed for non-ceria REO activity: (**left**) electrostatic attraction to negatively charged EPS and membranes, (**center**) phosphate complexation with biomolecular phosphate groups, and (**right**) ligand coordination with functional groups such as carboxylates and hydroxyls. These mechanisms shape REO adhesion, matrix interactions, and biological responses. (Created with BioRender.com; original artwork by the author).

For non-ceria REOs, the proposed mechanisms for their biological activity converge on a set of surface-mediated interactions rather than redox-driven pathways. These interactions include electrostatic attraction, phosphate complexation, and multidentate ligand coordination, each of which influences how REOs engage with extracellular polymeric substances (EPS), membranes, and biofilm matrices. Figure 3 illustrates these general interfacial processes, providing a conceptual framework for understanding the emerging and often material-dependent behaviors observed across samarium-, lanthanum-, and strontium-based systems.

### 4.1. Samarium Oxide: Functionalization-Dependent Antivirulence Activity

Samarium oxide (Sm_2_O_3_) has primarily been evaluated in functionalized or composite forms, making it difficult to determine its intrinsic antibiofilm capacity. Among these engineered systems, rutin-coated Sm_2_O_3_ nanoparticles exhibit the strongest activity, significantly inhibiting biofilm formation and suppressing virulence-factor expression, whereas uncoated Sm_2_O_3_ shows little to no measurable effect under comparable conditions [34]. Polyphenol coatings—including rutin—are increasingly recognized as potent modulators of metal-oxide surface chemistry and catalytic behavior, with recent work demonstrating that polyphenol–metal-oxide interfaces can enhance catalytic activity and biological function across diverse nanozyme systems [35]. This pattern suggests that the organic–oxide interface plays a major role in driving biological performance; however, because systematic studies on well-characterized, bare Sm_2_O_3_ nanoparticles are lacking, intrinsic antibiofilm properties cannot yet be determined.

Polyphenol coatings enhance nanoparticle interactions with EPS components and bacterial membranes and can interfere with quorum-sensing–regulated behaviors, producing antivirulence rather than bactericidal effects [34]. Additional evidence comes from samarium-doped hydroxyapatite coatings, which reduce biofilm formation while maintaining favorable biocompatibility profiles [36]. Together, these studies indicate that Sm-based antibiofilm effects arise primarily within engineered material systems, highlighting both the promise of functionalization and the clear need for systematic evaluation of unmodified Sm_2_O_3_.

### 4.2. Lanthanum Oxide: An Underexplored but Mechanistically Promising REO

Despite a strong biochemical rationale, La_2_O_3_ remains largely untested in direct antibiofilm nanoparticle studies. Lanthanum’s high phosphate affinity, used clinically in phosphate binders, suggests possible interactions with eDNA- and polysaccharide-rich EPS networks, but direct demonstration in mature biofilms is lacking. Indirect evidence from lanthanum-containing oxides and perovskite systems indicates possible membrane interactions and ion-homeostasis effects [37], yet these findings cannot be extrapolated to nanoparticulate La_2_O_3_ without experimental validation. The absence of data indicates that La_2_O_3_ remains an underexplored REO with plausible matrix-targeting capabilities. Practical factors, including the synthetic instability of nanoscale La_2_O_3_, rapid hydration to La(OH)_3_, and concentration-dependent cytotoxicity, may partially explain the limited literature and should be considered when assessing translational potential.

### 4.3. Strontium-Based and Mixed REO Systems

Strontium-containing oxides differ chemically from lanthanide REOs and operate through mechanisms that are not directly comparable to 4f-electron systems. SrO–CeO_2_ mixed oxides exhibit enhanced antibacterial and antibiofilm activity through complementary pathways, combining Ce-driven redox behavior with Sr-mediated ionic effects [38]. In contrast, strontium-containing bioactive glasses reduce *P. aeruginosa* biofilms primarily through ionic exchange and structural disruption rather than REO-like coordination or redox activity [39]. A recent review of green-synthesized SrO nanoparticles further shows that hybrid or composite SrO formulations consistently outperform isolated SrO, underscoring the importance of material architecture in determining activity [40]. Thus, while strontium-containing materials can act as synergistic components within composites, their mechanisms and classification differ fundamentally from classical REOs—an important distinction when comparing mechanistic frameworks.

Across samarium-, lanthanum-, and strontium-based systems, reported antibiofilm activity typically emerges within engineered materials, functionalized surfaces, or composite architectures. This pattern reflects the limited number of studies on unmodified REOs rather than a confirmed mechanistic constraint. Functionalization and composite design clearly enhance activity, but the intrinsic potential of non-ceria REOs remains insufficiently characterized to draw definitive conclusions. These observations help explain why CeO_2_ dominates the current literature while highlighting the need for systematic, mechanism-focused studies to clarify the true capabilities and limitations of non-ceria REOs.

## 5. Design Principles for Next-Generation REO Antibiofilm Materials

**Figure 4:**
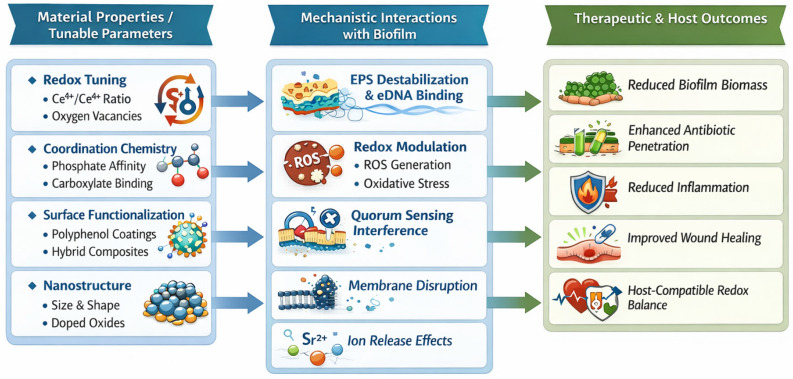
Design framework for REO antibiofilm materials. Material properties—including redox adaptability, coordination chemistry, nanostructure, and surface functionalization—determine how REOs engage with biofilm matrices through EPS destabilization, eDNA binding, redox modulation, quorum-sensing interference, and membrane interactions. These mechanistic processes collectively influence therapeutic outcomes such as reduced biofilm cohesion, improved antibiotic penetration, attenuation of inflammation, and enhanced tissue repair. The framework illustrates how engineered REO features translate into biological functions and guides future mechanism-informed REO design. (Created with BioRender.com; original artwork by the author).

The collective evidence across cerium-, samarium-, lanthanum-, and strontium-based systems demonstrates that antibiofilm activity in REOs is rarely an inherent property of the oxide lattice. Instead, it typically emerges from the interaction of multiple factors, including redox behavior, surface coordination chemistry, defect structure, and overall material architecture. CeO_2_ performs consistently well because it combines several of these features in a well-characterized manner [9,10,12], whereas non-ceria REOs have been explored far less extensively. As a result, they often appear active only when incorporated into functionalized, doped, or composite designs, not necessarily because these oxides lack intrinsic activity, but because the underlying mechanisms remain insufficiently studied. This landscape underscores the need for a unified design framework. To unify the mechanistic insights developed in earlier sections, Figure 4 presents a design framework linking REO material properties to biofilm-level interactions and resulting biological outcomes.

REO performance depends on deliberate tuning of composition, surface chemistry, defect density, and incorporation into composite or hybrid architectures, rather than on any assumed intrinsic antimicrobial property of the oxide phase. Through systematic adjustment of physicochemical parameters—such as surface charge, redox activity, ion-exchange behavior, and interfacial binding affinity—REO materials can be engineered to interfere with initial microbial adhesion, destabilize the extracellular polymeric matrix, and modulate quorum-regulated communication pathways.

Such engineered functionalities allow REOs to engage multiple antibiofilm mechanisms simultaneously, shifting the emphasis from direct bactericidal action toward matrix disruption, microenvironmental modulation, and synergy with co-therapies. These strategies also facilitate cooperative effects with antibiotics, natural products, biosurfactants, and other adjunctive agents, ultimately enhancing biofilm susceptibility while maintaining compatibility with host tissues.

### 5.1. Prioritize Redox Adaptability and Defect Engineering

CeO_2_’s prominence among REOs derives from its ability to cycle between Ce^3+^ and Ce^4+^ via oxygen-vacancy formation [11,12,21], enabling modulation of reactive oxygen species and disruption of biofilm redox homeostasis [8]. Future REO design may extend this behavior through mixed-valence oxides (e.g., Pr_6_O_11_ and Tb_4_O_7_), deliberate oxygen-vacancy engineering, and heterostructures that pair redox-active and redox-inactive phases. Such strategies aim to replicate CeO_2_-like adaptive catalytic responses, although direct evidence for the superior performance of engineered alternatives remains limited and should be investigated experimentally.

Representative examples include CeO_2_–La_2_O_3_ core–shell nanostructures, which enhance surface basicity and defect density, and SrO–CeO_2_ heterojunction oxides that couple Ce-mediated redox adaptability with Sr-driven ionic modulation. Additional systems, such as ceria–perovskite hybrid interfaces, illustrate how combining REO catalytic sites with perovskite-derived oxygen mobility can synergistically expand redox range and promote more efficient biofilm-matrix disruption.

These examples clarify the mechanistic logic behind heterostructure design, demonstrating how complementary physicochemical behavior can be integrated to surpass the functional limits of isolated oxide phases.

### 5.2. Exploit Coordination Chemistry for EPS Targeting

Because EPS cohesion depends on phosphate- and carboxylate-mediated crosslinking, REOs can disrupt matrix structure through strong coordination with these groups. Lanthanides with high charge density and strong Lewis acidity [18], such as La^3+^ and Sm^3+^, exhibit coordination tendencies that enable interactions with biomolecular ligands, although direct demonstrations of REO-mediated disruption of eDNA or polysaccharide components within EPS remain limited and largely inferred from model coordination studies [16]. Surface-modified REOs, including polyphenol-coated Sm_2_O_3_ formulations, show enhanced antibiofilm activity and increased association with matrix-rich environments [34]. Hybrid REO–polyphenol or REO–cationic polymer systems similarly display improved surface interactions and biofilm inhibition [36], yet detailed mechanistic mapping of REO–EPS interactions remains incomplete and is recognized as an open research area [15].

### 5.3. Integrate Functional Coatings to Unlock Antivirulence Pathways

Many non-ceria REOs (e.g., Sm_2_O_3_) have primarily exhibited antibiofilm or antivirulence activity in functionalized forms, reflecting the limited number of studies on bare oxides rather than a confirmed lack of intrinsic activity. Polyphenols such as rutin can convert poorly characterized REOs into antivirulence platforms by suppressing quorum-sensing-regulated behavior [34]. Functional coatings should therefore target communication, adhesion, or EPS synthesis, while the REO core provides stability and dispersibility, mirroring the success of rutin-coated Sm_2_O_3_ and offering a potentially generalizable strategy for other REOs.

### 5.4. Use Composite and Hybrid Architectures to Enhance Synergy

Strontium- and lanthanum-containing materials frequently show enhanced performance when incorporated into composite or hybrid architectures, including Sm-doped hydroxyapatite coatings [36], Sr-borate bioactive glass composites [39], and SrO–CeO_2_ mixed-oxide systems described in broader Sr-based nanomaterial reviews [38]. These formulations integrate multiple modes of action—ionic exchange, structural modification, redox modulation, and coordination-driven interactions—resulting in improved functional behavior compared with their isolated oxide counterparts. Reviews of green-synthesized SrO nanoparticles further indicate that hybrid or composite SrO systems consistently outperform single-component SrO, although the mechanistic basis for these enhancements differs substantially from those observed in lanthanide-based REOs [40].

### 5.5. Match REO Chemistry to Biofilm Microenvironmental Niches

Biofilms contain steep spatial gradients in oxygen, pH, redox state, nutrient availability, and EPS density [7], creating microenvironments that favor different REO chemistries. Redox-active oxides are well suited to hypoxic or ROS-buffering regions where redox modulation is advantageous [9]. Lanthanide REOs with high charge density and strong phosphate-binding tendencies may interact more strongly within eDNA-rich zones [16,18], while cation-releasing composites can be effective in regions where ionic crosslinking stabilizes the EPS matrix [39]. Surface-functionalized REOs with antivirulence or communication-disruption activity may preferentially affect quorum-sensing-intensive niches [34]. This spatially responsive design philosophy parallels CeO_2_’s well-documented ability to adjust its catalytic behavior according to local redox conditions [10].

### 5.6. Ensure Host Compatibility and Translational Feasibility

Biomedical REOs must balance antibiofilm efficacy with host safety. Key requirements include low cytotoxicity and compatibility with tissue repair processes [27,28], minimal inflammatory activation supported by nanomedicine studies [29,30], and sufficient stability in physiological environments to avoid premature transformation or clearance [23]. Practical deployment also depends on scalable and reproducible synthesis routes, as highlighted in recent reviews of green-derived REO composites [40].

Ceria succeeds because it combines redox adaptability, defect chemistry, and biomolecule affinity within a well-studied system. Other REOs may require engineering to achieve comparable functional profiles, though their full intrinsic potential has yet to be systematically characterized. A unified design framework for next-generation REO antibiofilm materials benefits from combining engineered redox activity, targeted coordination chemistry, functional coatings, composite architectures, microenvironment-specific deployment, and host-compatible performance—while acknowledging that rigorous comparative studies are still needed to map, which mechanisms are intrinsic, and which arise through deliberate material design.

## 6. Design Rules and Future Directions for REO-Based Antibiofilm Materials

The mechanistic convergence described above supports the formulation of design rules for REO antibiofilm materials. These principles aim to guide a shift from descriptive studies toward mechanism-informed material development. Importantly, they highlight conditions under which REO systems may succeed or underperform, without presuming that the lack of observed efficacy reflects intrinsic limitations of the underlying oxide. The evidence across cerium-, samarium-, lanthanum-, and strontium-based systems reveals that antibiofilm efficacy is an emergent property influenced by the combined control of redox behavior, surface chemistry, and material architecture.

### 6.1. Prioritize Matrix Destabilization over Bactericidal Potency

A central insight from REO antibiofilm studies is that effective biofilm disruption does not require complete bacterial killing. Mature biofilms resist bactericidal stress through EPS shielding, metabolic dormancy, and rapid recolonization. In contrast, REOs that weaken EPS cohesion—particularly through interactions with eDNA and charged polysaccharides—consistently reduce biofilm biomass and structural integrity [7,9]. This principle aligns with broader biofilm biology: matrix disruption restores susceptibility to antibiotics and host immunity even when bacterial viability is only partially affected.

REO formulations should be optimized for EPS and eDNA interactions, not maximal planktonic antimicrobial activity. Mechanical weakening, detachment assays, and EPS-specific endpoints should be prioritized alongside viability tests.

### 6.2. Redox Activity Must Be Tuned, Not Maximized

Redox modulation is a powerful antibiofilm mechanism, but excessive oxidative stress compromises host compatibility. CeO_2_ exemplifies a narrow redox window in which catalytic ROS modulation destabilizes biofilm redox homeostasis without inducing cytotoxicity [8,11,21]. This balance is essential for applications such as chronic wounds, where prolonged exposure is required. For redox-active REOs, controlling Ce^3+^/Ce^4+^ ratios, oxygen-vacancy density, and surface hydroxylation is more important than maximizing ROS generation. For REOs with limited intrinsic redox cycling, materials should be leveraged for their coordination- or interface-mediated interactions rather than artificially forced into oxidative modalities.

### 6.3. Surface Chemistry Is Decisive for Non-Ceria REOs

For REOs lacking intrinsic redox cycling, antibiofilm efficacy is strongly influenced by surface functionalization. Samarium oxide illustrates this dependency: antibiofilm and antivirulence activity has primarily been observed in ligand-modified systems that interfere with quorum sensing and biofilm maturation pathways [34]. Similar trends are observed in Sm-doped hydroxyapatite coatings, where dopants modulate surface charge and biomolecular affinity [36]. Given the limited data on unmodified non-ceria REOs, current evidence suggests that surface modification (e.g., polyphenols, peptides, cationic polymers, and EPS-binding motifs) enhances functionality, though more studies are needed to determine when such modification is essential versus beneficial.

### 6.4. Hybrid and Composite Systems Outperform Single-Component Oxides

In many reported cases, hybrid and composite materials containing lanthanum-, samarium-, or strontium-based components exhibit improved performance relative to isolated oxides, although mechanisms vary and the evidence remains uneven across systems. These multifunctional constructs leverage mechanistic complementarity—for example, pairing the redox adaptability of CeO_2_ with Sr-mediated ionic modulation or La-associated phosphate coordination [38,39]. Reported examples span strontium-borate bioactive glasses that disrupt EPS through ionic exchange, samarium-doped hydroxyapatite coatings that enhance surface reactivity and biocompatibility, REO–perovskite hybrids with strengthened catalytic and antimicrobial responses [37], and green-synthesized SrO nanocomposites that offer improved stability and reinforced EPS interactions [40]. These examples suggest that composite architecture can enhance stability, reactivity, or specificity, although determining when composites outperform single oxides requires further systematic comparison. Hybrid composites represent a promising direction, but not yet a proven universal requirement for effective REO antibiofilm performance.

### 6.5. Host Compatibility Is a Non-Negotiable Constraint

A recurring limitation of metal-based antibiofilm materials is host toxicity. CeO_2_’s success in chronic wound and diabetic ulcer models underscores the importance of biological permissiveness, particularly in applications requiring prolonged exposure [27,41]. Materials that collapse biofilms but damage surrounding tissue are unlikely to be translated clinically. Antibiofilm efficacy must be evaluated in parallel with host cell response, inflammatory signaling, and redox balance. REO systems that cannot maintain this balance may be more suitable for non-biomedical applications, depending on their toxicity profile.

### 6.6. Standardization and Model Selection Remain Critical Challenges

Comparisons across REO studies are often confounded by inconsistent biofilm models, endpoints, and exposure conditions. Prevention-focused assays are frequently misinterpreted as evidence of biofilm destruction and planktonic antimicrobial metrics remain overemphasized despite their limited relevance to mature biofilms. Future studies should clearly distinguish between biofilm inhibition, attenuation, and destruction and should employ mature biofilm models with standardized EPS, viability, and mechanical integrity metrics. Without such rigor, meaningful mechanistic comparisons will remain elusive.

REOs occupy a unique position at the intersection of materials science, microbiology, and biofilm biology. The evidence synthesized here supports viewing REOs as biofilm-modulating platforms that may restore susceptibility to antibiotics or host defenses rather than replace conventional antimicrobials. By applying the design principles outlined above, future work may transition from empirical screening toward predictive, mechanism-guided antibiofilm engineering.

## 7. Emerging Translational Evidence for REO-Mediated Biofilm Disruption

Although most rare-earth oxide (REO) antibiofilm studies remain preclinical, several clinical and near-clinical observations suggest that REO-based materials may disrupt biofilms in vivo. These examples reveal REOs’ value not as classical bactericidal agents, but as potential modulators of biofilm structure, microbial stress responses, and wound-healing trajectories. Their emerging clinical relevance lies in their hypothesized ability to shift chronic, biofilm-dominated environments toward productive tissue repair and reduced infection burden.

### 7.1. Diabetic Foot Ulcers: Emerging Clinical Evidence for Biofilm Destabilization

Clinical evidence supporting the antibiofilm potential of cerium oxide nanoparticles (CeO_2_ NPs) in diabetic foot ulcers (DFUs) remains extremely limited and primarily anecdotal, though early observations warrant cautious interest. The earliest documented clinical use involved topical CeO_2_ in neuropathic DFUs, where rapid improvements in wound appearance, reduced slough and exudate, diminished malodor, and fewer debridement requirements were observed [27]. Although biofilm biomass was not directly quantified, these nonspecific clinical improvements may be consistent with—but do not confirm—biofilm modulation, aligning with preclinical findings that CeO_2_ can weaken EPS cohesion and modulate redox stress in vitro.

Fu et al. [42] highlighted that CeO_2_ NPs address multiple pathological features of DFUs—oxidative stress, chronic inflammation, impaired angiogenesis, and dysregulated extracellular matrix remodeling—each deeply interconnected with biofilm persistence. CeO_2_’s ability to scavenge ROS through Ce^3+^/Ce^4+^ cycling may reduce oxidative damage and interrupt inflammatory loops that support biofilm stability. In addition, CeO_2_ NPs may modulate macrophage polarization, suppress NLRP3 inflammasome activation, and improve endothelial function—mechanisms that could indirectly influence biofilm resilience by restoring a healthier wound microenvironment. Notably, coated or hybrid CeO_2_ systems (polymer-coated, biomolecule-coated, or mesoporous-silica–hybrid CeO_2_) show superior stability, biocompatibility, and targeted activity compared with unmodified nanoparticles. These engineered systems enhance dispersion, reduce aggregation-related toxicity, and enable controlled release—properties theoretically relevant for chronic wounds. Several coated CeO_2_ platforms demonstrate improved antibacterial and antibiofilm performance in preclinical DFU models, including enhanced penetration into biofilm matrices and synergistic effects with antibiotics.

The isolated case reports and supporting preclinical data indicate only preliminary evidence that CeO_2_ NPs might help shift chronic diabetic wounds out of a biofilm-dominated inflammatory state. Robust conclusions await controlled clinical trials, and current evidence should be viewed as hypothesis-generating rather than confirmatory. Table 1 summarizes the principal biological functions attributed to CeO_2_ nanoparticles in diabetic wound studies. These mechanistic activities span antioxidant regulation, inflammatory modulation, macrophage polarization, antibacterial and antibiofilm effects, angiogenesis promotion, and extracellular matrix remodeling. The table integrates representative findings reported across studies [43,44,45,46,47,48,49,50,51,52,53,54,55,56,57,58,59,60,61,62,63,64,65,66,67,68,69,70,71,72,73,74,75,76,77,78,79,80,81] and highlights the multi-modal nature of CeO_2_-mediated wound repair.

Following the summary presented in Table 1, the mechanistic landscape of CeO_2_ nanoparticles in diabetic wounds becomes clearer when these functional categories are viewed as an interconnected biological cascade rather than isolated effects. The nanozyme-based antioxidant activity serves as the upstream driver, restoring redox balance in fibroblasts and endothelial cells and preventing the oxidative derailment that characterizes chronic diabetic wounds. This redox stabilization directly shapes the inflammatory milieu by suppressing NF-κB and NLRP3 signaling, lowering IL-1β, IL-6, and TNF-α levels, and reducing mitochondrial DNA leakage. In turn, this creates the conditions necessary for macrophage repolarization toward IL-4/IL-10-associated M2 phenotypes, enabling progression from a stalled inflammatory phase to a reparative one.

These immunometabolic shifts intersect with CeO_2_’s structural and antimicrobial contributions, including weakening of EPS matrices, improved antibiotic penetration, and enhanced control of biofilm-associated bacterial persistence. Downstream, CeO_2_ supports angiogenesis through HIF-1α (hypoxia-inducible factor-1 alpha) stabilization, VEGF (vascular endothelial growth factor) upregulation, and protection of endothelial cells from mitochondrial ROS. These processes converge on extracellular matrix remodeling, where fibroblast activation, collagen deposition, and MMP-9 (matrix metalloproteinase-9) modulation collectively restore dermal architecture. Viewed holistically, the studies summarized in Table 1 [43,44,45,46,47,48,49,50,51,52,53,54,55,56,57,58,59,60,61,62,63,64,65,66,67,68,69,70,71,72,73,74,75,76,77,78,79,80,81] position CeO_2_ as a systems-level wound-modulating platform, which is capable of addressing the biochemical, immunological, microbial, and structural deficits that define diabetic ulcers.

### 7.2. Nanozyme-Based Cryogels in Infected Diabetic Wounds: A Mechanistic Analog

Nanozyme-based cryogels accelerate healing in infected diabetic wounds through coordinated biofilm disruption and anti-inflammatory activity [28]. Although these cryogels are not REO-based, their catalytic ROS-modulating behavior parallels key features of CeO_2_ nanozymes, providing a conceptual—rather than material—analog relevant to REO translation. In vivo, this system substantially reduced *P. aeruginosa* biofilm biomass, restored redox balance within the wound bed, enhanced epithelialization and collagen deposition, and lowered inflammatory cytokine expression. These outcomes support a broader mechanistic principle: catalytic nanomaterials capable of modulating ROS flux and interacting with wound-bed matrices may benefit chronic, biofilm-dominated wounds regardless of their elemental composition.

### 7.3. Orthopedic and Dental Implants: Lessons from REO-Doped Biomaterials

Samarium-doped hydroxyapatite coatings reduce biofilm formation in vitro [36], aligning with the clinical need to prevent early implant colonization. Since implant infections arise rapidly—often from *S. aureus* or *S. epidermidis*—materials that alter surface adhesion or early microbial communication may reduce early biofilm establishment. Although clinical testing of REO-doped coatings is still pending, their preclinical mechanism of action and osseointegration potential position them as promising but unvalidated candidates.

### 7.4. Bioactive Glass Composites: Strontium-Based Systems in Wound Care

Strontium-containing bioactive glass composites have shown antibiofilm activity against *P. aeruginosa* [39]. Because bioactive glass dressings are already approved clinically, these findings suggest a potential translational route, although additional REO-specific validation remains needed. The combination of ionic exchange, pro-regenerative effects of Sr^2+^, and tunable dissolution profiles aligns well with chronic wound needs.

### 7.5. Pathways to Clinical Adoption

Based on current evidence, the most realistic near-term applications for REO antibiofilm materials fall into three practical domains. The first is chronic wound care, where REOs can be incorporated into hydrogels, scaffolds, or sprayable dressings, most commonly CeO_2_, but also Sr- or Sm-doped bioactive glass formulations and catalytic nanozyme composites. A second emerging area is implanting protection, using Sm-doped hydroxyapatite, La- or Sr-modified perovskite coatings, and CeO_2_-based thin films to reduce biofilm formation on device surfaces. The third involves adjunctive therapeutic strategies, where REOs enhance antibiotic efficacy, support combination debridement gels, or participate in catalytic and photodynamic approaches designed to weaken biofilms and resensitize pathogens.

Recent studies across metal-oxide and nanozyme platforms further contextualize the unique antibiofilm potential of REOs. Comprehensive analyses of rare-earth oxide nanoparticles highlight their emerging therapeutic relevance in multidrug-resistant infections and wound environments, where mixed-valence redox cycling and oxygen-vacancy–mediated catalysis enable adaptive modulation of oxidative stress [82]. Complementary mechanistic insights from hybrid catalytic systems—such as nitric-oxide–releasing photothermal nanozymes—demonstrate how multimodal nanomaterials can simultaneously disrupt biofilms, modulate redox balance, and accelerate tissue regeneration, providing functional analogs to REO nanozyme behavior without substituting for it [83]. Classical metal and metal-oxide nanomaterials provide an important contrast point, as their antimicrobial activity is dominated by direct ROS generation, ion release, and membrane disruption rather than environment-responsive catalysis [84]. Within REO systems, synergistic combinations with antibiotics have shown promising enhancement of bacterial clearance and biofilm suppression, underscoring the potential of REOs as adjunctive agents rather than standalone antimicrobials [85]. Mechanical disruption strategies—such as magnetically actuated iron-oxide nanoparticles that physically break biofilm matrices—illustrate an orthogonal design space that may be integrated with REO-based catalytic approaches in future hybrid systems [86]. These cross-platform insights reinforce the broader mechanistic landscape in which REO nanozymes operate and highlight opportunities for designing multifunctional antibiofilm materials that combine catalytic, physicochemical, and mechanical modes of action. These applications exploit REOs’ potential strengths, matrix disruption, redox modulation, and host compatibility. However, evidence remains preliminary, and translation will require rigorous safety evaluation, standardized biofilm endpoints, and controlled clinical studies. CeO_2_ and certain other metal-oxide nanozymes can generate controlled ROS levels, influencing EPS stability or microbial physiology. Such effects are highly dose-dependent and context-dependent and may alternatively protect bacteria under some conditions (e.g., CeO_2_–H_2_O_2_ interactions).

The Zhu et al. [87] finding is now contextualized that CeO_2_ nanoparticles exhibit peroxidase-like activity that can attenuate certain oxidative antibacterial mechanisms, as Zhu et al. [87] demonstrated by showing that CeO_2_ can protect bacteria from H_2_O_2_-mediated killing. However, this does not undermine CeO_2_’s wound-healing potential, because its primary therapeutic actions in DFUs appear host-directed (anti-inflammatory, antioxidant, and pro-angiogenic).

### 7.6. Biosurfactant-Based Synergy

Biosurfactant-based and surface-engineered antimicrobial systems offer important parallels for understanding how REOs may be enhanced through interfacial design. Plant-derived or biologically synthesized REO composites illustrate this concept clearly, as arctium lappa–synthesized CeO_2_ nanoparticles encapsulated in nano-chitosan disrupt microbial adhesion and suppress biofilm maturation while maintaining biocompatibility, demonstrating how organic coatings can augment REO–biofilm interactions [88]. REO-enabled surface engineering also extends to anti-adhesive nanocomposites. High-throughput–synthesized CeO_2_ nanocrystals incorporated into transparent polymer matrices repel *P. aeruginosa* biofilms by modifying nanoscale surface energy and topography, indicating that REO surfaces can inhibit early-stage attachment even without chemical killing [89]. Complementary mechanistic studies show that CeO_2_ nanozyme mimics interfere with redox-regulated quorum-sensing pathways, producing measurable disruption of microbial communication networks and weakening coordinated biofilm responses [90]. However, REO interactions are not universally synergistic. CeO_2_ and Fe_3_O_4_ nanoparticles have been shown to abolish ciprofloxacin activity against both Gram-positive and Gram-negative biofilm-forming bacteria, likely due to ROS scavenging, drug adsorption, or altered microenvironmental chemistry [91]. This aligns with broader ESKAPE pathogen biology, which emphasizes the importance of antivirulence strategies over classical antibiotic potentiation [92].

Insights from metal oxide–biosurfactant systems provide an additional design direction. Biosurfactant-coated silver and iron oxide nanoparticles, as well as rhamnolipid-coated ZnO, display enhanced anti-adhesive and antibiofilm properties through reductions in surface tension, improved wettability, and disruption of EPS-mediated attachment [93,94,95]. Reviews of biosurfactants underscore their ability to modulate surface interactions, interfere with microbial communication, and reduce matrix cohesion—mechanisms that may be transferable to REO platforms [96,97]. Finally, computational and machine-learning studies offer predictive tools for designing REO–biosurfactant hybrids. Advances in materials-focused machine learning that accelerate discovery and enable structure–property mapping [98,99], together with progress in molecular modeling [100,101], nanozyme–enzyme interaction simulations [102], and machine-learning–based toxicity prediction [103], provide a coherent path toward the rational optimization of surface coatings, catalytic behavior, and safety profiles. These insights suggest that integrating biosurfactant-inspired design principles with REO chemistry represents a promising, underexplored route to achieving multi-modal, host-compatible antibiofilm materials.

### 7.7. Antibiotic-Class-Specific Mechanisms of REO Synergy

Beyond general synergy mechanisms, REO interactions with antibiotics also show clear class-dependent distinctions, which are important for interpreting therapeutic combinations. REO–antibiotic interactions vary substantially across antibiotic classes. For aminoglycosides, REO-mediated weakening of EPS structure and localized changes in membrane charge distribution can facilitate deeper penetration of cationic drugs and improve ribosomal access.

Synergy with quinolones likely arises from REO-induced modulation of redox-responsive stress pathways, which can sensitize biofilm-embedded cells to DNA-targeting mechanisms by disrupting protective redox homeostasis. In contrast, interactions with β-lactams appear to involve REO-driven alterations in cell-wall stress responses and reduced EPS shielding, which can increase the vulnerability of peptidoglycan remodeling pathways to β-lactam inhibition. Although detailed datasets remain limited, these distinctions clarify that REO–antibiotic combinations do not operate through a single unified mechanism but through class-dependent pathways shaped by EPS architecture, redox physiology, and microbial stress adaptation.

These mechanistic differences reinforce the need to evaluate REO–antibiotic combinations within antibiotic-specific physiological contexts rather than assuming uniform potentiation across drug classes.

## 8. Regulatory, Safety, and Manufacturing Considerations

REOs show promise as antibiofilm agents, but clinical translation hinges on regulatory classification, safety, and manufacturing control. Their physicochemical stability and catalytic activity offer therapeutic advantages yet pose challenges for risk assessment and quality assurance. Regulatory classification depends on intended use—REO systems may be regulated as devices, drugs, or combination products. Many REOS act via physicochemical mechanisms, aligning more with device or hybrid categories. This demands rigorous characterization of particle size, morphology, surface chemistry, aggregation, dissolution, biodistribution, and clearance. Mechanistic descriptors like oxygen-vacancy density and valence stability are relevant but not yet standardized.

Safety remains a bottleneck. Ceria shows encouraging biocompatibility, but other REOs may generate ROS, release cations, or trigger immune responses depending on surface charge, oxidation state, or coating degradation. Chronic-use candidates require immunological profiling and long-term studies of organ accumulation and clearance. Toxicology data for most REOs are limited, underscoring the need for systematic evaluation. Manufacturing adds complexity as small variations in synthesis, valence distribution, defect density, and coating uniformity can alter biological outcomes. Scaling production while ensuring batch consistency is challenging. GMP (Good Manufacturing Practices)-grade pipelines for REO nanomaterials are still emerging. Additional concerns include environmental impact, occupational safety, and patient acceptance. Life-cycle assessments and environmental fate studies are increasingly emphasized by regulators. Successful translation will require coordinated advances in mechanistic understanding, toxicology, regulatory strategy, and manufacturing standards. Ceria leads the field, while non-ceria REOs may need functionalization or composite design to match their safety and consistency.

## 9. Future Research Priorities

REOs offer promising antibiofilm potential, but their advancement requires coordinated progress in mechanistic modeling, material engineering, translational integration, and safety evaluation.

### 9.1. Standardized Mechanistic Biofilm Models

Most REO studies rely on early-stage assays that overlook mature biofilm complexity. Future models should incorporate EPS-rich, multi-day systems with pathogens like *P. aeruginosa*, *S. aureus*, and *Candida* spp., and include endpoints such as rheology, detachment force, and eDNA content. These standardized mechanistic models provide the foundation for evaluating REO performance against clinically relevant pathogens, including the ESKAPE group discussed in Section 9.9.

### 9.2. Systematically Evaluate Non-Ceria REOs

Oxides of samarium, lanthanum, and strontium show promise, but mechanisms remain unclear. While CeO_2_ dominates REO antibacterial studies [8,9,10,11,12], comparatively fewer investigations have evaluated La_2_O_3_ [16], Sm_2_O_3_ [34,36], or Sr-based oxides [38,39,40], underscoring the need for systematic comparisons. Mixed-valence systems (e.g., Pr_6_O_11_ and Tb_4_O_7_) should be benchmarked under standardized conditions. A curated REO library would enable meaningful comparisons.

### 9.3. Engineer REO–Organic Hybrids

Functionalized REOs often outperform bare oxides. Incorporating quorum-sensing inhibitors, EPS-targeting ligands, or cationic peptides may enhance catalytic behavior and broaden activity.

### 9.4. Develop Composite Architectures

Doped and hybrid systems—such as ceria–strontium composites, REO–perovskite hybrids, and nanozyme–REO constructs—enable synergistic effects like redox modulation and EPS destabilization, though comparative data remain limited.

### 9.5. Integrate into Clinical Platforms

Embedding REOs into hydrogels, wound dressings, nanofiber mats, and implant coatings will clarify real-world performance under biofilm-dominated conditions.

### 9.6. Advance Safety and Toxicology

Long-term studies on organ retention, immune activation, and oxidative stress thresholds are essential. Most REOs lack comprehensive toxicology data, limiting safe-by-design development.

### 9.7. Leverage Predictive Modeling

Machine learning and molecular simulations can accelerate REO optimization by predicting structure–activity relationships and toxicity risks [98,99,100,101,102,103].

### 9.8. Foster Interdisciplinary Collaboration

Progress depends on bridging materials science, microbiology, wound care, computational modeling, and regulatory science. REOs sit at the intersection of these domains, requiring integrated ecosystems for clinical translation.

### 9.9. Targeting ESKAPE Biofilms with REOs

ESKAPE pathogens (*Enterococcus faecium*, *Staphylococcus aureus*, *Klebsiella pneumoniae*, *Acinetobacter baumannii*, *Pseudomonas aeruginosa*, and *Enterobacter* spp.) represent the most clinically challenging biofilm-forming organisms in chronic wounds. Their persistence is driven by dense EPS matrices, efflux-mediated antibiotic tolerance, metabolic dormancy, and quorum-regulated defense systems. As highlighted in recent analyses of non-thermal plasma strategies for ESKAPE control [104], effective disruption of these biofilms requires agents capable of simultaneously weakening the matrix, perturbing redox homeostasis, and interfering with microbial communication networks. REOs are well-positioned to meet these criteria. Although Scholtz et al. [104] evaluated non-thermal plasma rather than REOs, it is included here as a mechanistic benchmark because it identifies the multi-modal disruption strategies required to overcome mature ESKAPE biofilms—matrix weakening, redox imbalance, and interference with quorum-regulated defense systems. These same mechanistic requirements are directly relevant for evaluating the potential of REO-based materials.

In this context, future studies should explicitly evaluate REO performance against mature ESKAPE biofilms under clinically relevant conditions. Key priorities include determining whether CeO_2_ and non-ceria REOs can (i) destabilize EPS architectures characteristic of *P. aeruginosa* and *A. baumannii*, (ii) modulate redox-responsive defense pathways in *S. aureus* and *Enterococcus* spp., (iii) enhance antibiotic penetration into carbapenem-resistant *Klebsiella* and *Enterobacter* biofilms, and (iv) suppress quorum-regulated virulence circuits. Comparative studies with established anti-biofilm modalities—such as non-thermal plasma—would clarify whether REOs can serve as complementary or alternative platforms for ESKAPE control. Establishing these capabilities would significantly expand the translational relevance of REOs, positioning them as versatile agents capable of addressing the most recalcitrant biofilm-associated infections.

## 10. Conclusions

REOs are emerging as versatile antibiofilm materials, with cerium oxide remaining the benchmark due to its redox adaptability, oxygen-vacancy–driven catalysis, and strong host compatibility. Yet the broader REO family—samarium, lanthanum, strontium, and mixed-valence systems—holds underexplored potential when strategically engineered through surface modification, functional coatings, or composite architectures. Across studies, a unifying theme is clear: effective biofilm disruption is not an intrinsic property of any oxide but an engineered outcome that depends on aligning REO physicochemical behavior with biofilm architecture, including EPS cohesion, redox gradients, and quorum-sensing networks.

Early preclinical evidence suggests REO-based materials may modulate chronic wound microenvironments and influence infection trajectories, though controlled clinical trials remain absent. Their value lies less in replacing antibiotics and more in resensitizing pathogens, attenuating virulence, and supporting tissue repair. Realizing this potential will require standardized biofilm models, rigorous mechanistic toxicology, scalable manufacturing, and sustained interdisciplinary collaboration. By integrating redox engineering, coordination chemistry, and functional surface design, REO systems can evolve from empirical constructs into predictable, clinically relevant antibiofilm technologies. Rather than viewing REOs solely as antimicrobial agents, they should be recognized as adaptable biofilm-modulating platforms capable of contributing to next-generation strategies for managing chronic infections.

## Figures and Tables

**Table 1:** Representative functions of CeO_2_ nanoparticles in diabetic wound studies (Table created by the author based on mechanistic findings reported in studies [43,44,45,46,47,48,49,50,51,52,53,54,55,56,57,58,59,60,61,62,63,64,65,66,67,68,69,70,71,72,73,74,75,76,77,78,79,80,81]).

Therapeutic Role	Representative Mechanistic Findings	References
Antioxidant activity	SOD-, CAT-, and POD-like behavior; reduction of intracellular ROS in fibroblasts and endothelial cells; protection of redox-sensitive cellular pathways	[43,44,45,46,48,50,51]
Anti-inflammatory effects	Suppression of NF-κB and NLRP3 inflammasome activation; decreased IL-1β, IL-6, and TNF-α; mitigation of mitochondrial DNA leakage	[54,55,56,57,58,59,60,61]
Macrophage polarization	Promotion of IL-4/IL-10-associated M2 reparative macrophages; enhancement of inflammation-resolution pathways	[63,64,65,66]
Antibacterial/antibiofilm effects	Weakening of EPS matrices; improved antibiotic penetration; observed synergy in CeO_2_–ciprofloxacin systems	[67,71]
Angiogenesis promotion	Stabilization of HIF-1α; increased VEGF expression; reduced mitochondrial ROS; enhanced endothelial survival	[72,73,74,75,76,77]
ECM remodeling	Enhanced fibroblast activity and collagen deposition; modulation of MMP-9 levels; improvement of dermal structural integrity	[78,79,80,81]

## Data Availability

No new data was generated in this review.
